# Knowledge of diabetes risk factors and preventive measures among attendees of a primary care center in eastern Saudi Arabia

**DOI:** 10.4103/0256-4947.51813

**Published:** 2009

**Authors:** Abdullah S. Aljoudi, Attia Z. A. Taha

**Affiliations:** a From the Department of Biostatistics, Epidemiology and Scientific Computing Department, Research Centre, King Faisal Specialist Hospital and Research Centre, Riyadh, Saudi Arabia; b From the Family and Community Medicine Department, College of Medicine, King Faisal University, Al-Khobar, Saudi Arabia

## Abstract

**BACKGROUND AND OBJECTIVE:**

Few studies have been conducted in Saudi Arabia to assess the level of awareness and knowledge of the population about diabetes mellitus (DM) risk factors and preventative measures. The objective of this study was to measure this knowledge among attendees of a primary care center in eastern Saudi Arabia.

**METHODS:**

A sample of 300 male and female Saudis aged 18 years and older from the catchment area of the Aqrabya Primary Care Center were randomly selected for this cross-sectional survey. Data were collected through a structured face-to-face interview using a pre-piloted Arabic instrument. Regression analysis was used to identify the predictors of knowledge.

**RESULTS:**

The 288 participants interviewed included 100 males and 188 females. The mean (SD) age was 44.7 (12.6) years for males and 33.8 (12.4) years for females. Fewer than 50% of participants knew about DM risk factors and preventive measures. In a regression model that included age, sex and education, education had a statistically significant positive association with knowledge of risk factors (odds ratio 12.5, 95% CI 6.26-25.2, *P*<.001) and preventive measures (odds ratio 7.6, 95% CI 4.01-14.2, *P*<.001), and age had a statistically significant negative association with knowledge of DM risk factors (odds ratio 0.377, 95% CI 0.207-0.685, *P*=.001) and prevention (odds ratio 0.407, 95% CI 0.231-0.717, *P*=.001). The main risk factor stated by participants was obesity (35.8%), while the main preventive measure mentioned was weight reduction (37.9%).

**CONCLUSION:**

Attendees had poor knowledge of DM risk factors and preventive measures. The level of education and age were important predictors of knowledge. Programs for health education of the community about DM risk factors and preventive measures are needed.

Diabetes mellitus (DM) is an escalating public health problem with an estimated global prevalence of 2.8% in 2000 and a projected prevalence of 4.4% in 2030 (171 million in 2000 to 366 million in 2030).^1^ Saudi Arabia, a country undergoing a rapid epidemiologic transition,^2^ is witnessing a steady increase in the prevalence of DM 3–6 with the most recent estimate of prevalence being as high as 23.7% among adult citizens.^6^

Despite the fact that the cause of DM is unknown, many of its modifiable lifestyle-related risk factors have been identified and studied. The accumulating evidence suggests that DM is a potentially preventable disease if its risk factors are identified early and avoided.^7^–^10^ Lifestyle interventions (e.g. physical activity, weight loss) have proven to be more effective than medicine in preventing or delaying the onset of DM in persons at high risk of developing the disease.^7^ However, transferring such evidence into an effective community intervention program requires an understanding of the specific needs of these communities before introducing any kind of intervention.^11^–^13^ Learning about DM risk factors and preventive measures is the first step in prevention, since it will enable the public to make the informed decision of adopting a healthy lifestyle.^11^,^14^ In addition, policy makers as well as public health practitioners need reliable and valid data regarding the distribution and determinants of DM-related health issues among their population. These data are needed to design, implement and evaluate successful intervention programs.^12^

In Saudi Arabia in general, and in Al-Khobar in particular, there are few studies conducted to assess the level of awareness and knowledge of the population of DM risk factors and preventative measures. Since such knowledge forms the basis for the development of community intervention programs, this study was conducted to provide DM-related information to the health care team at the Aqrabya Primary Care Center. Such information will help in designing, implementing and evaluating a health education program about risk factors and prevention of DM for the attendees of this center. The specific objective of the study was to assess the awareness and knowledge of attendees of the Aqrabya Primary Care Center about DM risk factors and preventive measures.

## METHODS

This cross-sectional study population consisted of adult Saudi males and females (ages 18 years and above) attending the Aqrabya Primary Care Center, the largest center in Al-Khobar, Eastern Saudi Arabia. The center is the first health contact point for all those living in the catchment area and had a catchment population of 53616 in the year 2005.

The sample size (n) was calculated using the following formula:^15^

n=[DEPF*Np(1-p)]/[(d2/Z21-α/2*(N-1)+p*(1-p)]

Where N is the population size=53616, p the prevalence of DM(=24%±5%), d the confidence limits (=5%), DEFF the design effect (=1) in this case. The above formula yielded a sample of 279, which was rounded for practical reasons to 300. The sample was selected using probability sampling with proportional allocation of gender representation in the study population. As the ratio of male to female in the study population was 1:2, 100 males from the male reception and 200 females from the female reception were selected using a systematic random sampling technique. Given 22 working days per month and an average of 200 persons attending the center daily, the number of participants to be interviewed per day was 300/22=14. The random interval for the 14 participants was 200/14=14. For practical purposes, every 15th attendee of the center was invited to participate in the study. Selection of the first participant was done randomly using a random number table from which a number between 1 and 14 was selected. For example, if number 9 was chosen randomly on the male side then the first participant would be the 9th attendee to the male reception desk at that day; then the next participant would be the 24th, 39th, 54th and 69th accordingly. Similarly, in the female side if number 14 was chosen randomly then the first participant would be the 14th attendee to the female reception desk at that day; then the next participants would be the 29th, 44th, 59th, 74th, 89th, 104th, 119th, 134th, and 149th accordingly. If a selected attendee (e.g. the 89th) was not eligible to participate in the study (i.e. non-Saudi or under age of 18 years) or refused to participate, then the next attendee (e.g. the 104th) would be invited to participate. For practical reasons, the study was conducted over a period of 20 days by recruiting 10 female and 5 male attendees on an average each day as long as the number of attendees for that day allowed. For example, if the number of attendees on a certain day allowed for recruiting more than five participants on the male side and more than ten on the female side then the sampling procedure would countinue to recruite as many participants as the number of attendees allowed, but if the number of attendees on a certain day led to recruiting less than five on the male side and less than ten on the female side then this would be compensated for by recruiting more participants in the coming days.

To ensure validity, data was collected through a structured face-to-face interview with participants using a pre-piloted Arabic instrument, instead of distributing a self-administrated questionnaire among participants. The instrument was piloted by interviewing volunteers at the family medicine clinic of the university to test the clarity of questions and to estimate the time needed to complete an interview. All questions were asked directly in a standardized way to ensure data reproducability (reliability). The instrument included demographic questions (i.e. age, sex, educational status) and open-ended questions about DM risk factors (i.e. What might lead to diabetes?), as well as prevention (i.e. What might prevent diabetes?). Trained interviewers conducted the interviews; the first author interviewed male participants and a female nurse interviewed female participants. Participants who had attended one or more formal schooling establishments were defined as educated, while others were defined as undereducated. Knowledge of DM risk factors and preventive measures were coded 0 for not mentioning correctly any risk factor or preventive measure and 1 for mentioning correctly at least one risk factor or one preventive measure.

OpenEpi (version 2)^15^ and EpiInfo (version 3.3.2)^16^ were used for data entry and analysis. Frequency distributions were obtained and regression analysis was used to identify the predictors of knowledge. The chi-square and *t* tests were used to test significance, with a *P* value <.05 indicating statistical significance; 95% confidence intervals were also calculated.

The study was approved by the research ethics committee at the Department of Family and Community Medicine and permission to conduct the study was granted by the Director of Aqrabya Primary Care Center. The objective of the study was explained to the participants and their verbal consent was acquired before conducting the interview. Health education pamphlets on the risk factors and prevention of DM were provided to all participants at the end of the interview.

## RESULTS

Of the 300 sampled attendees, 288 (response rate, 96%) agreed to participate in the study, including 100 (34.7%) males and 188 (65.3%) females. Mean (SD) for age was 44.7 (12.6) years for males and 33.8 (12.4) years for females (t=7.06, df=286, *P*<.001). Other characteristics are shown in Table 1. Overall, 121 participants (42.0%) had knowledge of DM risk factors and 120 (41.7%) had knowledge of DM prevention. Forty-four males (41%), 51 (38.1%) subjects 40 years of age or older and 96 (59.6%) educated participants had knowledge of DM risk factors. There was a statistically significant association between educational status and knowledge of risk factors (chi-square=46.5, df=1, *P*<.001). Forty-four males (44%), 50 (37.3%) subjects 40 years of age or older and 92 (57.1%) educated participants had knowledge of DM prevention (Table 2). There was a statistically significant association between educational status of participants and knowledge of DM prevention (chilsquare=35.98, df=1, *P*<.001) (Table 3). Of three independent variables (age, sex, and education) entered into a regression model, education had a significant positive association with knowledge of DM risk factors and prevention, while age had a significant negative association with knowledge of DM risk factors and prevention (Table 4). The risk factors of DM most frequently stated by respondents were obesity and lack of physical exercise (Table 5). The most commonly mentioned preventive measures were weight reduction and exercise. Of all participants, 77 (26.7%) stated two or more DM risk factors and 89 (30.9%) mentioned two or more preventive measures (Table 5).

**Table 1:** Characteristics of the respondents in a primary care center in eastern Saudi Arabia, 2006 (n=288).

Variable	No. (%)	95% Confidence interval
Age (years)		
<40	154 (53.5)	47.5-59.3
≥40	134 (46.5)	40.7-52.5

Sex		
Male	100 (34.7)	29.2-40.5
Female	188 (65.3)	59.5-70.8

Education		
Educated	161 (55.9)	50.1-61.5
Undereducated	127 (44.1)	38.5-49.9

Knowledge of risk factors		
Yes	121 (42.0)	36.5-47.8
No	167 (58.0)	52.2-63.5

Knowledge of prevention		
Yes	120 (41.7)	36.1-47.4
No	168 (58.3)	52.6-63.9

**Table 2:** Knowledge of DM risk factors in relation to age, sex and education among the respondents (n=288).

Variables	Knowledge of DM risk factors		Total No (%)	Chisqaure test (df)	*P*
	Yes No (%)	No No (%)			
Age (years)				1.608 (1)	.205

<40	70 (45.5)	84 (54.5)	154 (100)		
≥40	51 (38.1)	83 (61.9)	134 (100)		
**Total**	**121 (42.0)**	**167 (58.0)**	**288 (100)**		

Sex				0.065 (1)	.799

Male	41 (41.0)	59 (59.0)	100 (100)		
Female	80 (42.6)	108 (57.4)	188 (100)		
**Total**	**121 (42.0)**	**167 (58.0)**	**288 (100)**		

Education				46.493 (1)	<.001

Educated	96 (59.6)	65 (40.4)	161 (100)		
Undereducated	25 (19.7)	102 (80.3)	127 (100)		
**Total**	**121 (42.0)**	**167 (58.0)**	**288 (100)**		

**Table 3:** Knowledge of diabetes prevention in relation to age, sex and education among the respondents (n=288).

Variables	Knowledge of DM prevention		Total No (%)	Chisqaure test (df)	*P*
	Yes No (%)	No No (%)			
Age (years)				1.954 (1)	.162

<40	70 (45.5)	84 (54.5)	154 (100)		
≥40	50 (37.3)	84(62.7)	134 (100)		
**Total**	**120 (41.7)**	**168 (58.3)**	**288 (100)**		

Gender				0.343 (1)	.558

Male	44 (44.0)	56 (56.0)	100 (100)		
Female	76 (40.4)	112 (59.6)	188 (100)		
**Total**	**120 (41.7)**	**168 (58.3)**	**288 (100)**		

Education				35.978 (1)	<.001

Educated	92 (57.1)	69 (42.9)	161 (100)		
Undereducated	28 (22.0)	99 (78.0)	127 (100)		
**Total**	**120 (41.7)**	**168 (58.3)**	**288 (100)**		

**Table 4:** Regression analysis showing predictors of knowledge of diabetes risk factors and prevention among the respondents (n=288).

Variables	Knowledge of diabetes risk factors			Knowledge of diabetes prevention		
	Odds ratio	95% CI	*P*	Odds ratios	95% CI	*P*
Age (<40=0)*	0.377	0.207-0.685	.001	0.407	0.231-0.717	.001
Education (Undereducated-0)^a^	12.548	6.259-25.158	<.001	7.558	4.010-14.243	<.001

a Reference category; CI: Confidence intervals

**Table 5:** Diabetes risk factors and preventive measures as stated by the respondents (n=288).

Knowledge	No. (%)	95% Confidence interval
Risk factors		
Obesity	103 (35.8)	30.5-41.5
Lack of physical exercise	93 (32.3)	27.2-37.9
Smoking	81 (28.1)	23.3-33.6
Genetic	33 (11.5)	8.3-15.7
Two or more of the above	77 (26.7)	21.9-32.1
Others (e.g. stress, alcohol, drugs)	44 (15.3)	11.6-19.9

Preventive measures		
Reduce weight	109 (37.9)	32.4-43.6
Perform physical exercise	91 (31.6)	26.5-37.2
Two or more of the above	89 (30.9)	25.9-36.5
Others (e.g. avoid stress, take medicine)	38 (13.2)	9.8-17.6

## DISCUSSION

The lack of knowledge of risk factors of a disease (DM in this case) may impede preventive efforts such as the adoption of positive lifestyle changes. Therefore, a knowledgelbased perception of personal risk for the disease appears to be an important factor in many preventive health behaviors.^17^,^18^

In this study more than half of the participants were not able to correctly mention any DM risk factors or preventive measures. This lack of knowledge could be explained by educational status. The association between education and knowledge in this study is consistent with the results from several studies in Saudi Arabia and in other cultures.^19^–^23^ Taha and Bella (1998),^19^ who explored the knowledge of causes and prevention of coronary heart diseases (CHD) in a primary care setting in eastern Saudi Arabia found that knowledge increased with higher level of education. Mosca et al (2004)^20^ reported a similar association in the American Heart Association Study as did Louise et al,^21^ who stated in their Canadian population survey that: “the strongest and most consistent association was between education and knowing risk factors.” The React Study in Europe,^22^ a community-based survey designed to study the public perception of cardiovascular risk in five European countries, reported a similar association. Rafique and Khuwaja^23^ conducted a survey in Pakistan to assess public awareness about diabetes, hypertension and lifestyle and found that the level of knowledge was positively associated with educational status. A recent community-based study conducted in Oman to evaluate knowledge and perception of diabetes in the general population reported a similar positive association between educational status and knowledge of DM risk factors and prevention and lack of knowledge of DM risk factors and prevention.^24^ An interesting result in this study was the negative association between knowledge and age. Younger participants were more likely to have knowledge of DM risk factors and prevention than older participants, which is consistent with the findings of Louise et a1.^21^

In our study, obesity was the most commonly mentioned risk factor (35.8%), which is comparable with other studies.^19^–^23^ Taha and Bella^19^ in their Al-Khobar-based study, reported that 17.6% of the participants mentioned obesity as a risk factor for CHD. Even though fewer than half of the participants in this study were able to mention any risk factor or preventive measure of DM, most of them reported correctly the most important modifiable risk factors, namely obesity and lack of physical exercise. Similarly the most important preventive measures, namely weight reduction and physical exercise were mentioned. The improvement in the knowledge of participants in this study in comparison to Taha's study may be due to the 10-year gap between the two. Since that time, a major public awareness campaign for DM has taken place in the study setting.^25^ The Prince Mohammad Bin Fahad Campaign for DM was launched in 2004, a wide community-based campaign conducted on 214381 participants and aimed at screening for DM and hypertension. It was accompanied by local media coverage in public places (e.g. malls), where health education materials were distributed to the public as well as participants. While the earlier study did not attempt to explore the source of knowledge of the participants, it is possible that the improvement in knowledge as measured by this study could, in part, be attributed to this mass campaign.

We may have underestimated the level of knowledge, as a limitation of having used unaided open-ended questions within the questionnaire as opposed to mentioning specific risk factors. However, we believe that this question type allowed for identification of the most known DM risk factors and preventive measures. Several findings from this study support the need for well-designed health education programs at the community level for primary prevention of DM.^11^ The program should address each persons unique situation and cater to interpersonal variation, and should include the local needs of the older and undereducated population in particular.
